# The American College of Radiology white paper on radiation dose in medicine:deep impact on the practice of cardiovascular imaging

**DOI:** 10.1186/1476-7120-5-37

**Published:** 2007-10-31

**Authors:** Eugenio Picano, Eliseo Vano, Richard Semelka, Dieter Regulla

**Affiliations:** 1 CNR, Institute of Clinical Physiology, Pisa, Italy; 2 Complutense University, San Carlos University Hospital, Madrid, Spain; 3 University of North Carolina at Chapel Hill, Dept of Radiology, Chapel Hill, NC, USA; 4 GSF-National Research Center for Environment and Health, Institute for Radiation Protection, Neuherberg, Germany

## Abstract

In April 2007, the American College of Radiology released the "White Paper on Radiation Dose in Medicine". The Blue Ribbon panel members included private practice and academic diagnostic radiologists, medical physicists, representatives of industry and regulatory groups, and a patient advocate. The panel concluded that the expanding use of imaging modalities using ionizing radiations such as CT and nuclear medicine may result in an increased incidence of radiation-related cancer in the exposed population in the not-too-distant future, and this problem can likely be minimized by preventing the inappropriate use of such imaging and by optimizing studies that are performed to obtain the best image quality with the lowest radiation dose. The White Paper set forth practical suggestions to minimize radiation risk, including education for all stakeholders in the principles of radiation safety and preferential use of alternative (non-ionizing) imaging techniques, such as MRI and ultrasound. These recommendations are especially relevant for cardiologists, who prescribe and/or practice medical imaging examinations accounting for at least 50% of the total effective dose by radiation medicine, which amounts to an equivalent of about 160 chest x-rays per head per year in US. Were they be enacted, these simple recommendations would determine a revolution in the contemporary way of teaching, learning and practising cardiology.

## Radiation in cardiology

**Figure 1:**
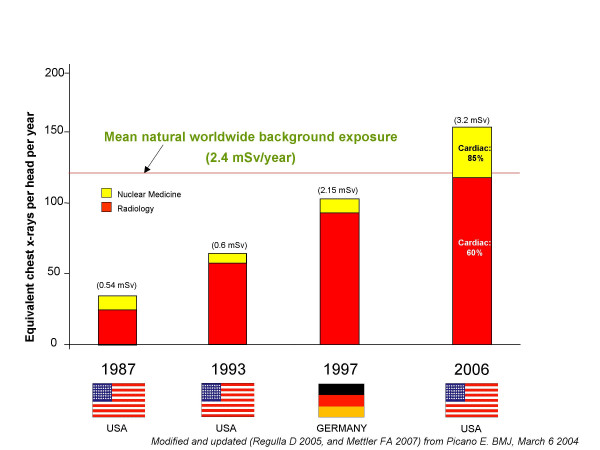
Medical and natural sources of radiation. Modified from ref 1, and updated with 2006 data from ref. 3. The effective dose of 1 mSv is equivalent to 50 chest x-rays. The per-head dose of ionizing radiation from clinical imaging exams in the United States increased almost 600 percent from 1980 to 2006.

The medical use of radiation is the largest man-made source of radiation exposure. About 5 billion imaging examinations are performed worldwide each year, and 2 out of 3 employ ionizing radiations with radiology or nuclear medicine [1]. In the developed countries, exposure from medical ionizing test results in a mean effective dose per year per head in the range of 100 (Germany, radiological year 1997) [2] to 160 chest x-rays (USA, radiological year 2006) [3] – an amount higher than that originating from one year of natural background radiation: Fig. 1. With now obsolete radiological dose estimates, referred to 1991–1996 and excluding nuclear medicine exposures, Berrington and Darby estimated in 2004 that 0.6 (for UK) to 3.2% (for Japan) of cancers could be caused by diagnostic x-rays. The attributable cancer risk from diagnostic x-rays was 0.9% for USA and 1.5% for Germany [4]. In 1991–96, the mean exposure for the US citizen was 0.5 mSv per head per year from x-rays. In 2006, the estimated exposure (from radiology and nuclear medicine) reaches an unprecedented 3.2 mSv per head per year (more than 6-fold higher) than the estimate used by Berrington. The attributable cancer risk will rise accordingly – at least around 5% risk of cancers from diagnostic radiation [5]. The inappropriateness of imaging techniques with high doses and high long-term risks is economically and socially unsustainable, but it also opens a unique opportunity to abate healthcare costs, reduce long-term risks, and improve health care standard simply targeting inappropriate examinations.

Cardiologists prescribe and/or directly perform >50% of all imaging examinations, accounting for about two thirds of the total effective dose to patients [3,6]. Mettler et al recently reported data referred to the radiological year 2006 in USA. There were almost 20 million studies of nuclear medicine. Cardiac studies account for 57% of all nuclear medicine studies and 85% of the dose [3]. Bedetti et al reported data referred to modern adult cardiological patients, who receive a median cumulative effective dose of 60 mSv per head [6]. Three types of procedures were responsible for about 86% of the total collective effective dose: 1) arteriography and interventional cardiology (12% of examinations, 48% of average dose per patient); 2) nuclear medicine (5% of examinations, 21% of average dose per patient; 3) CT (4% of examinations, 17% of average dose per patient). Conventional X-ray examinations represent 79% of total number of examinations corresponding only to 14% of collective effective dose [6]. Radiological dose estimate can be expressed as multiples of a single postero-anterior chest x-ray (equal to 0.02 milliSievert, mSv), as originally suggested by the UK College of Radiologists and endorsed in the European Commission referral guidelines on medical imaging [7,8]. The radiological dose estimate of common cardiological examinations may range from an equivalent of about 600 chest x-rays of a stress scintigraphy with sestamibi to about about 1,500 chest x-rays for a Thallium scan. The dose of a 64-slice cardiac Computed Tomography corresponds to about 750 chest x-rays, the dose of a coronary angiography and stenting to about 1,000 chest x-rays [7-11]: Table 1. Unfortunately, cardiologists (and even radiologists) show little awareness of the dose of the exam they daily perform or request [7,12,13]. Also as a consequence of the lack of radiological awareness, the rate of inappropriate examinations is unacceptably high in cardiology, even for procedures with high radiation load and a non-negligible long-term cancer risk, such as stress perfusion scintigraphy [14] and coronary angiography [15]. The high, dominant cardiological component of the exposure of patients and the high, unprecedented professional exposure of interventional cardiologists (three times higher than radiologists) [16] are the two main reasons of the growing interest of the cardiology community towards the radiation issue [17]. Now, the release of the landmark White Paper of the American College of Radiology [18] gives impetus to the need for the cardiological community to also address the subject. A "white paper" (so called because it was originally bound in white) is an authorative report on a major issue, as by a team of experts. It was written by a "blue ribbon committee", i.e., an independent commission of non-partisan experts formed to investigate some important governmental issues. The following summarizes many of the important issues from that publication.

**Table 1:** Doses in cardiology

**Examination**	**Effective dose (mSv)**	**Equivalent n. of chest x-rays**
**CONVENTIONAL RADIOLOGY**		
♣ Chest x ray (single postero-anterior)	0.02	1
**NUCLEAR MEDICINE**		
♣ Tc-99 m tetrafosmin cardiac rest-stress (10 mCi+30 mCi)*	10.6	530
♣ Tc-99 m sestamibi cardiac 1-day rest-stress (10 mCi+30 mCi)*	12	600
♣ Tc-99 m sestamibi cardiac 2-day stress-rest (30 mCi+30 mCi)*	17.5	775
♣ Tl-201 cardiac stress and reinjection (3.0 mCi+1.0 mCi)*	25	1500
♣ Dual isotope cardiac (3.0 mCi Tl201 + 30 mCi Tc-99 m)*	27	1600
**64-Slice CARDIAC COMPUTED TOMOGRAPHY**		
♣ ECG pulsing, no aorta**	9	450
♣ No ECG pulsing, yes aorta**	29	1450
**INTERVENTIONAL RADIOLOGY**		
♣ Conventional rhythm device***	1.4	70
♣ Cardiac resynchronization device***	5.5	275
♣ Cerebral angiography ***	1.6–10.6	80–530
♣ Coronary angiography ***	3.1–10.6	155–555
♣ Abdomen angiography ***	6–23	300–1150
♣ Peripheral angiography***	2.7–14	135–700
♣ Coronary angioplasty ***	6.8–28.9	340–1445
♣ Peripheral angioplasty***	10–12	500–600
♣ Radiofrequency ablation***	17–25	850–1250
♣ Valvuloplasty***	29	1450

From ref. 8, 9*, 10**,11 ***. CT protocols that rescan the same region of interest (e.g., non-contrast and contrast-enhanced scans) impart two to three times the radiation dose.

### White paper lesson number 1: low dose ionizing radiation is a proven carcinogen

**Figure 2:**
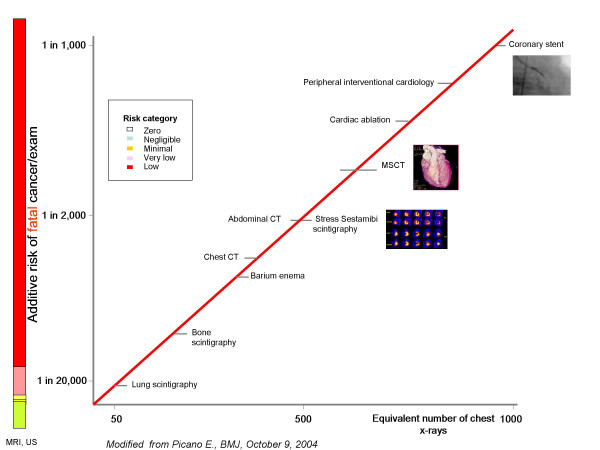
Graphical presentation of cancer risk and radiation dose (in multiples of exposure from a conventional chest x-ray exam) for some common cardiovascular examinations. Modified from ref. 7, on the basis of novel estimates of BEIR VII (ref. 21).

Ionizing radiation has long been known to increase the risk of cancer. In fact, x-rays and γ-rays have recently been officially classified as "carcinogen" by the World Health Organization's International Agency for Research on Cancer [19], the Agency for Toxic Substances and Disease Registry of the Centers for Disease Control and Prevention [20], and the National Institute of Environmental Health Sciences [21]. Because radiation is a relatively weak carcinogen, it is difficult to isolate radiation-induced cancer. According to the updated risk estimates released in the recently Seventh Report of the authorative Committee to Assess Health Risks from Exposure to Low Levels of Ionizing Radiation (BEIR VII report), the attributable risk of cancer is 1/750 for 15 mSv exposure, corresponding to the dose estimate of a coronary MSCT; 1/500 for 20 mSv exposure, corresponding to the dose estimate of a coronary stent; 1/400 for 25 mSv exposure, corresponding to the dose estimate of a Thallium scan) [22]: Fig 2. *"Does this mean that current radiation exposure can be neglected? The answer is no. Radiation-induced cancers typically do not occur until 1 or 2 decades or longer after exposure. Thus, any increase in cancer occurrence due to the burgeoning medical exposures in the part 2 decades, as is the case for CT and nuclear medicine studies, may not be expected to be evident for many years" *[18].

### White paper lesson number 2: physicians have the duty to know what they do

The present generation of cardiologists can no longer afford to be ignorant of the risks related to medical radiation exposure [7,12,13]. As the White Paper elegantly puts it, *"although some referring physicians are very knowledgeable regarding safety issues and incorporate such information into their imaging decisions, others have had little or no training in radiation exposure and do not routinely consider this factor when ordering imaging examinations" *[18]. Simply, because of the dramatic increase in the number of diagnostic examinations performed each year, we have to be more cognizant of the long-term cancer risk in the risk-benefit assessment of new technologies [8,11].

### White paper lesson number 3: patients have the right to know what they do

**Figure 3:**
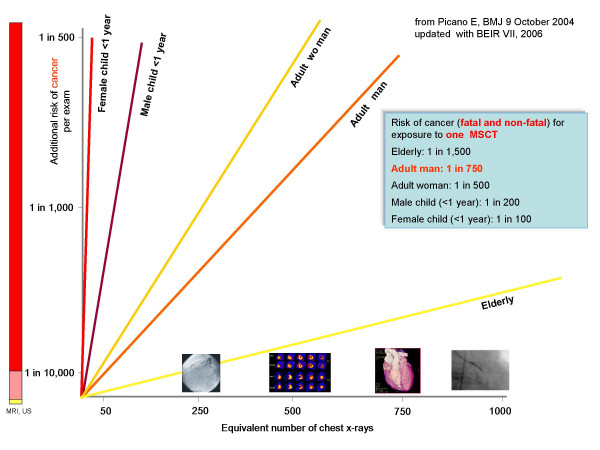
Risk stratified according to age and gender. The risk is 37% higher in women than in men, and 4-fold higher in children <1 year than in adults. The risk is reduced by one-half in elderly (>80 years). Redrawn and modified from ref. 7, on the basis of novel estimates of BEIR VII (ref. 21).

Recent data clearly show that patients are largely unaware of the dose (and the long-term risk) of the imaging studies they undergo [23,24]. In particular, patients undergoing common cardiac imaging examinations involving significant exposure have little knowledge about radiological exposure (and corresponding risk) [13]. Patients obviously have the right to know, according to common sense, medical deontological code, and the law [7]. The White Paper gently puts it as follows [18]: *"Radiologists understand the potential dangers from ionizing radiation far better than patients do, yet not every radiologist provides a balanced assessment of the risks and benefits of imaging when patients undergo testing. It is incumbent on radiologists to assume the responsibility for their patient's safety with regard to radiation exposure. They should also educate their patients on these issues so they may make informed decisions about their health care. Although patients frequently want to know the radiation "dose" they will receive during examinations, they are generally unfamiliar with radiation technology and may not understand the level of risk" *[18]. The graph of radiation risk communication [7], updated with BEIR VII estimates [22], may serve to the purpose of risk-dose communication: Fig. 2. This radiation risk graph was also endorsed and suggested by the Italian Institute of Health and Ministry of Health as a way respectful of patients' rights to communicate radiation risk [25]. Risk is highest in small children (Fig. 3), but unfortunately also paediatricians [23] and paediatric cardiologists [12] have very little awareness of these risks.

### White paper lesson number 4: change cardiology teaching

Cardiologists intensively use radiology, and therefore they must know radiobiology and radioprotection essentials. In our curriculum, our meetings, our textbooks, our scientific journals, the radiation information is typically absent or presented in an esoteric, clinically irrelevant way [7]. The White Paper suggests a profound remodelling of radioprotection teaching: *"Educating future referring physicians on radiation exposure to patient during diagnostic imaging must begin during medical school. The method of instruction, clerkship or general curriculum is not as important as the goal of inculcating the awareness of radiation exposure in students during training. By prominently displaying the relative radiation exposure level for a particular examination during order entry [in the electronic format of physician order entry system], a clinician may be steered toward an imaging regimen that minimizes radiation"*. It is true that medical physicists often are involved in cardiology fellowship training, especially in invasive cardiology. However, the White Paper states, and we agree, they must "*present their courses clearly, concisely and in a clinical relevant manner. Such an initiative will hopefully result in resident's discovering that instruction in medical physics is interesting and memorable, so that they will subsequently be able to apply the principles learned throughout their careers*" [18]. The cultural benefit of this remodelling of training will be immense to enhance the protection of patients and physicians themselves [26].

### White paper lesson number 5: More clinical research on radiation biology and genetics is needed

**Figure 4:**
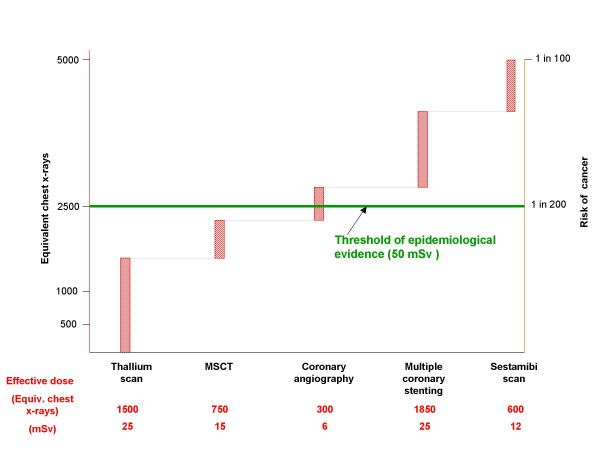
The cumulative exposure of doses (y axis, left) and corresponding risk (y axis, right) with a standard, radiation-insensitive, diagnostic algorhythm for coronary artery disease. In the x-axis, we listed some common cardiologic examinations with the corresponding doses (1 mSv = 50 chest x-rays). The threshold of 50 mSv of epidemiological evidence is surpassed by a typical cardiologic patient with known or suspected coronary artery disease, in one single hospital admission.

From the clinical point of view the calculation of cancer risk for radiation-exposed patients is presently based on the "linear-no threshold model", i.e. on the assumption that no safe dose exists; the higher the dose, the greater the risk [4]. At doses in excess of 50 mSv, equivalent to 2,500 chest X-rays or one whole-body CT without contrast, with re-scan after contrast [18], the approximately linear increase of cancer risk with dose has been directly observed by epidemiological research. In cardiology, it is likely to surpass the dose of 50 mSv [6], even in a single hospital admission for a single problem, most commonly a suspicion of coronary artery disease. Fig. 4 illustrates the cumulation of the doses due to five radiological examinations undergone in a typical case. At doses below 50 mSv, i.e. in most cases of radiological patient exposure (see fig. 2 and 3), epidemiological data are not available, and an extrapolation of cancer risk from higher to lower doses has to be performed, although on the expense of accuracy, in the light of radiobiological knowledge. More data are needed, e.g. to better understand the genetic, immunologic and environmental factors modulating low dose radiation damage. The White Paper states: "*Many questions remain unanswered regarding the fundamental mechanisms of radiation injury. Deoxyribonucleic acid breakage, chromosomal aberrations, and gene mutations caused by radiation exposure, as well as the potential for deoxyribonucleic acid to repair itself between radiation exposures, are important avenues for further investigation*". The existence of many unknowns, however, does neither justify the neglect of the possibility of cancer risk nor the restraint in putting up the warning sign. Therefore the International Commission on Radiological Protection has recommended to use the linear-no threshold model only for prospective risk estimates even in this low dose range [27]. Barrington de Gonzalez and Darby [4] have shown how to do this, not concealing the existing lack of precision of such estimates. In this situation, much more research work is needed, such as DNA and chromosomal biodosimetry studies for pediatric [28] and adult [29] cardiological patients and cardiology professionals exposed to radiation in the workplace [30]. A small, but important, sign of the growing interest of cardiological community towards radiation issues is that the largest Cardiology Association in Italy endorsed and funded a project on "Effects of chronic low dose radiation exposure on reproductive health on interventional cardiologists" [31]. Cardiologists start exploring the "dark side of the moon", the unwanted effects of radiation exposure, knowing that this is one of the ways – and probably not the least important – to be a good doctor.

### White paper lesson number 6: remodel current cardiology practice

Learning all the previous lessons will lead to an obvious change in cardiological practice. At present, we often think in terms of acute risk versus acute diagnostic benefit. There is no doubt that acute benefits of cardiac imaging are immense. However, an exam that considers only acute benefits may be less desirable when one considers also the long-term risks in the use of ionizing radiation-base imaging, specifically cancer induction. Ignoring long-term risks, it has been reported that up to one-third of cardiac stress scintigraphies [14] and up to 50% of coronary angiographies [15] are unnecessary; which, for completeness, is also observed with highly specialized non-ionizing examinations such as stress echocardiography [32]. The White Paper outlines the future scenario with potential to change this very worrying situation: *"There should be special attention paid to the practical suggestions set forth in this paper, such as education for all stakeholders in the principles of radiation safety, the appropriate utilization of imaging to minimize any associated radiation risk, the standardization of radiation dose data to be archived during imaging for its ultimate use in benchmarks good practice, and, finally, the identification and perhaps alternative imaging of patients who may have already reached threshold levels of estimated exposure from diagnostic imaging"*. These straightforward recommendations, if enacted in the cardiovascular community, will radically change the way cardiology is learned, taught, and practised today. It will be aparadigm shift in medical imaging: from benefit to risk-benefit [33-35], as recommended by good radiological protection since long, although also not so strictly adhered to by radiologists [36,37]. To illustrate this with cardiac stress imaging, 10 million cardiac scintigraphies per year can be, in theory, replaced by an approach based on stress echo and on stress cardiac-MRI – deemed to be equally effective by specialist guidelines [33]. Small individual risks, even if their magnitude is not known precisely, undergone in million examinations are likely to become significant population risks. To achieve the goal of sustainability [1], the radiation issue should be considered a shared problem of everyone involved in patient-care and communication with the public through the lay press [38], and will require the joint efforts of physicians, specialists, patients, vendors, clinical governance authorities, and politicians [18].

A recent clinical competence statement of interventional cardiologists accept that "responsibilities on all physicians is to minimize the radiation injury hazard to their patients, to their professional staff, and to themselves" [39]. Coronary interventionalists – but, probably, all cardiologists – "must have a thorough knowledge of consequences of exposure of patients and personnel to ionizing radiation, and methods of reducing patient and staff radiation exposure" [40]. In the words of the High Commissioner of the US Nuclear Regulatory Commission, "*if you listen to newsradio here in Washington, every morning you will hear advertisement from heart scans, full body scans, any scans you can think of for asymptomatic patients. Of course, they do not advertise you're getting rems as you get these scans. They do not advertise radiation at all*" [41]. Also in scientific cardiological meetings and articles, radiation is often "not advertised at all" and very little space in our journals is devoted to radiation issues. This situation is likely to change in the very near future.

We have summarized American College of Radiology landmark White Paper on Radiation Dose in Medicine, and heartily endorse it. The next step is for the Cardiology community to take stock in their practice of medical imaging, and move toward a more patient-focused approach emphasizing patient safety, especially in regards to ionizing radiation and the long-term risks of cancer.
